# Association of Handgrip Strength and Muscle Mass with Dependency in (Instrumental) Activities of Daily Living in Hospitalized Older Adults -The EMPOWER Study

**DOI:** 10.1007/s12603-019-1170-5

**Published:** 2019-02-19

**Authors:** C.G.M. Meskers, E.M. Reijnierse, S.T. Numans, R.C. Kruizinga, V.D. Pierik, J.M. van Ancum, M. Slee-Valentijn, K. Scheerman, S. Verlaan, Andrea B. Maier

**Affiliations:** 1 Department of Human Movement Sciences, @AgeAmsterdam, Amsterdam Movement Sciences, Amsterdam, The Netherlands; 2 Department of Rehabilitation Medicine, Amsterdam UMC, Amsterdam, The Netherlands; 3 Department of Medicine and Aged Care, @AgeMelbourne, Royal Melbourne Hospital, University of Melbourne, Melbourne, Australia; 4 Department of Internal Medicine, Section of Gerontology and Geriatrics, Amsterdam UMC, Amsterdam, The Netherlands; 5 Center of Excellence in Geriatric Rehabilitation, Cordaan, Amsterdam, The Netherlands; 6 @Age, Department of Human Movement Sciences, Amsterdam, Movement Sciences, Van der Boechorststraat 9, 1081 BT, Amsterdam, The Netherlands

**Keywords:** Muscles, muscle mass, muscle strength, handgrip strength, activities of daily living, hospitalization, outcome assessment, aged

## Abstract

**Objectives:**

Handgrip strength (HGS) and muscle mass are strong predictors for dependency in Activities of Daily Living (ADL) and Instrumental Activities of Daily Living (IADL) in community dwelling older adults. Whether this also applies to older hospitalized patients is yet unknown. We studied the association between HGS and muscle mass with ADL and IADL dependency at admission and change of ADL and IADL dependency at three months after discharge in older hospitalized patients.

**Design:**

Observational longitudinal inception cohort (EMPOWER) including 378 patients aged 70 years and older.

**Setting:**

Four different clinical wards of a university teaching hospital, The Netherlands.

**Measurements:**

HGS and muscle mass were measured within 48 hours after admission using hand dynamometry and Bio-electrical Impedance Analysis respectively. ADL dependency was assessed using the Katz score (0-6 points) and IADL dependency using the Lawton and Brody score (0-8 points) within 48 hours after admission and three months after discharge.

**Results:**

At admission, lower HGS was associated with ADL dependency in both males and females. Lower muscle mass was associated with ADL dependency in males. Lower HGS was associated with IADL dependency, but only in males. Lower HGS at admission in males was associated with an increase in ADL dependency three months after discharge.

**Conclusion:**

In hospitalized older patients, HGS is associated with ADL and IADL and muscle mass measures with ADL in male patients only. HGS should be explored as predictive marker for outcome of hospitalized older patients after discharge.

## Introduction

Hospitalization rates among older adults account to more than one third of hospital admissions (1). Hospitalization in older adults is associated with poor health outcomes such as cognitive decline (2), as well as increased dependency in Activities of Daily Living (ADL) (3) and Instrumental Activities of Daily Living (IADL) (4), which in turn can lead to decreased autonomy and decreased quality of life and even increased mortality (5).

Low muscle strength and low muscle mass are diagnostic measures of sarcopenia and are prevalent in up to 45% of acutely hospitalized patients aged 65 years or older (6, 7). In community-dwelling older adults, low muscle strength and low muscle mass are associated with increased dependency in ADL (8), incident falls (9, 10) and mortality (11). Hospitalization frequently goes along with immobilization (5) and induces a decrease in muscle strength and muscle mass in electively admitted patients (12). Low muscle strength and low muscle mass could have prognostic value for poor health outcome after hospitalization and could possibly be a target for intervention in hospitalized patients (13). To the best of our knowledge, it is unknown whether muscle strength and/or muscle mass have prognostic value for dependency in (instrumental) activities of daily living after hospital discharge.

The aim of this study was to determine the association of muscle strength and muscle mass at admission with 1) ADL and IADL dependency at admission and 2) change in ADL and IADL dependency from admission to three months after discharge in hospitalized older patients aged 70 years and older.

## Methods

### Study design

The ‘Evaluation of Muscle parameters in a Prospective cohort of Older patients at clinical Wards Exploring Relations with bed rest and malnutrition’ (EMPOWER) study is an observational, prospective inception cohort study. A total of 838 subsequent hospitalized patients were screened for eligibility for inclusion from April 2015 to December 2015. Patients were included if they were aged 70 years and older and admitted to one of four clinical wards (acute admission, internal medicine, neurosurgery and orthopedics, traumatology) of the VU University Medical Center, Amsterdam, The Netherlands, a middle-sized teaching hospital. Patients were excluded if they were terminally ill, had an expected length of stay in the hospital shorter than 24 hours, were admitted at another hospital and relocated to the VU University Medical Center, were nursed in isolation rooms or were not able to understand the Dutch language. The EMPOWER study included a total of 378 patients. Patients were assessed within 48 hours after hospital admission, comprising assessment of patient characteristics, measurements of muscle strength and muscle mass, and ADL and IADL dependency. ADL and IADL dependency was re-assessed at three months after discharge using telephone calls as described earlier (14). This study was reviewed and approved by the medical ethical committee of the VU University Medical Center, Amsterdam, The Netherlands. Informed consent was obtained from all included patients.

### Characteristics of patients

At admission, the following items were assessed during an interview by a nurse: living situation, use of walking aid, risk of malnutrition defined by the Short Nutritional Assessment Questionnaire (SNAQ) score using a score ≥2 out of 7 points (15), risk of delirium (16), cognitive impairment defined by the Six Item Cognitive Impairment Test (6CIT) score using a score ≥11 out of 28 points (17).

The following items were collected from medical records: age, sex, multimorbidity, polypharmacy, acute or elective admission and length of stay. Multimorbidity was defined as two or more of the following diseases: cerebrovascular accident/transient ischemic attack, chronic obstructive pulmonary disease, hypertension, heart failure, chronic kidney disease, diabetes mellitus, malignancy, peripheral vascular disease, thyroid disease, Parkinson’s disease, arthrosis, rheumatoid arthritis and osteoporosis. Polypharmacy was defined as five or more medications (18).

Weight was measured to the nearest 0.1 kilograms using a weighing chair. Self-reported weight was used when measurement of weight was impossible. Knee height was measured to the nearest 0.1 centimeter to estimate actual height to the nearest 1 cm using the Longitudinal Aging Study Amsterdam (LASA) formula (19). BMI was calculated to the nearest 0.1 kilogram/meter2 as weight divided by height squared.

### Handgrip strength

HGS was measured using a hydraulic hand dynamometer (Jamar, Sammons Preston Rolyan, IL, USA). Measurements were performed according to instructions recommended by the American Society of Hand Therapists (20), i.e. patients were asked to sit straight up in a chair or on the edge of the bed, flex their elbow about 90 degrees, not support their elbows and squeeze maximally. If patients were unable to sit, patients were asked to put the bed in an angle of approximately 30 degrees, flex their elbow about 90 degrees, not support their elbows and squeeze maximally under verbal encouragement. Two measurements per hand side alternately were performed. The maximal value of all four measurements was used for analysis.

### Muscle mass

Body composition was measured using direct segmental multi-frequency bioelectrical impedance analysis (DSM-BIA) (InBody S10, Biospace Co., Ltd, Seoul), which is a validated and standardized method of estimating skeletal muscle mass compared to DXA (21). Patients were asked to lie down in bed, spread their arms to about a 15 degrees’ angle, arms not touching the trunk and to spread legs to shoulder width, thighs not touching each other. If patients were not able to lie down flat, measurements were performed in maximum supine position that could be reached. BIA measurements were not performed when the patient had a pacemaker or other implanted electronic device (n = 25) and when electrodes could not be placed on fingers or ankles (n = 21). The following measures of muscle mass were used for analyses: skeletal muscle mass (SMM) in kilograms, SMM as a percentage of total body weight (SMM%) (8) and skeletal mass index as SMM divided by height squared (SMI) (22). Fat mass in kilograms was obtained during measurement of body composition and was measured to the nearest 0.1 kilograms.

### Activities of daily living and instrumental activities of daily living

ADL dependency was assessed using the Katz score (23) ranging from 0 to 6 points, a lower score meaning greater dependency. For the cross-sectional analysis, ADL score was dichotomized: a score of <6 points was considered dependent and a score of 6 points was considered independent (24). IADL dependency was determined using the Lawton and Brody score (25) ranging from 0 to 8 points, a lower score meaning greater dependency. IADL score was dichotomized: a score of <8 points was considered dependent and a score of 8 points was considered independent (26). Due to a protocol amendment at a later stage in which the assessment of IADL was added to the protocol, IADL data at admission are available in sample of 262 (69.3%) patients.

For longitudinal analysis, the change in ADL and IADL was defined as the score at follow-up assessment minus the score at admission (change in ADL and IADL); positive change ADL and IADL scores indicating a decrease in dependency and negative change in ADL or IADL scores indicating an increase in dependency.

### Statistical Analysis

Continuous variables were presented as mean with standard deviation (SD) if normally distributed and as median with interquartile range (IQR) if not normally distributed.

Cross-sectional analysis of the associations of HGS, SMM, SMM% and SMI (independent variables) with ADL and IADL dependency (dependent variables) at admission were analyzed using binary logistic regression analysis (independency = 1, dependency = 0). In model 1, analyses were adjusted for age. In model 2, analyses were additionally adjusted for multimorbidity and weight (kg) and height (m) (for HGS) or fat mass (kg) (for SMM and SMI) or weight (kg) (for SMM%) (27).

Longitudinal analyses of the association of HGS, SMM, SMM% and SMI with the change in ADL and IADL scores were analyzed using linear regression analyses. In model 1, analyses were adjusted for age and ADL or IADL score at admission. In model 2, analyses were additionally adjusted for multimorbidity and weight (kg) and height (m) (for HGS) or fat mass (kg) (for SMM and SMI) or weight (kg) (for SMM%) (27).

Statistical analyses were performed using the Statistical Package for the Social Sciences, version 22 (IBM SPSS Statistics, IBM Corporation, Chicago, IL). P-values lower than 0.05 were considered statistically significant. P-values lower than 0.1 were considered a trend.

## Results

Table 1 shows the patient characteristics at admission. The mean age of the total population was 79.7 years (SD 6.43) and 192 (50.8%) patients were male. Most patients (n=320, 84.7%) were acutely admitted. HGS and muscle mass were lower in females compared to males. ADL and IADL dependency and scores at admission and three months post-discharge are shown in Table 2. ADL scores did not significantly change from admission to three months discharge. IADL scores were significantly lower at three months discharge compared to admission indicating relative increase of dependency in females and males.

**Table 1:** Patient characteristics at admission

	**N**	**Females n=186**	**Males n=192**
Age, years	378	80.3 (6.54)	79.1 (6.23)
Living independently, n (%)	373	161 (89.0)	178 (92.7)
Use of walking aid, n (%)	375	108 (58.8)	92 (48.2)
Risk of malnutrition^a^, n (%)	367	56 (30.8)	68 (36.0)
Risk of delirium, n (%)	375	110 (60.1)	114 (59.4)
Cognitive impairment^b^, n (%)	370	37 (20.3)	28 (14.9)
Multimorbidity^c^, n (%)	376	163 (88.6)	170 (88.5)
Polypharmacy^d^, n (%)	340	92 (55.1)	116 (67.1)
Acute admission, n (%)	378	153 (82.3)	167 (87.0)
Length of stay, median [IQR]	377	5.13 [3.05-8.77]	4.88 [2.69-7.34]
Weight, kg	378	68.7 (18.3)	77.4 (14.6)
Height^e^, cm	378	162 (6.27)	175 (6.85)
BMI, kg/m^2^	378	26.4 (6.82)	25.2 (4.41)
Fat mass, kg	321	25.7 (12.5)	21.7 (9.49)
HGS, kg	378	14.9 (5.65)	26.1 (9.84)
SMM, kg	321	22.5 (3.76)	29.8 (5.62)
SMM%, %	321	33.7 (5.66)	39.1 (5.00)
SMI, kg/m^2^	321	8.64 (1.24)	9.70 (1.48)


All variables are presented as mean (SD), unless otherwise indicated. IQR: interquartile range, BMI: body mass index, HGS: handgrip strength, SMM: skeletal muscle mass, SMI: skeletal muscle mass index. ^a^Short Nutritional Assessment Questionnaire score ≥2. ^b^Six Item Cognitive Impairment Test ≥11. ^c^Number of medications ≥5. ^d^Number of comorbidities ≥2. ^e^Based on knee height and LASA formula.

**Table 2:** Activities of daily living and instrumental activities of daily living at admission and three months post-discharge

	**Admission**			**Three months post-discharge**			**Change**	
	**N**	**Dependency, n (%)**	**Score, median [IQR]**	**N**	**Dependency, n (%)**	**Score, median [IQR]**	**N**	**Score, mean (SD)**
ADL								
Females	181	97 (53.6)	5 [3-6]	151	87 (57.6)	5 [4-6]	149	0.21 (1.78)
Males	192	91 (47.4)	6 [3-6]	140	64 (45.7)	6 [4-6]	140	0.16 (1.69)
IADL								
Females	131	77 (58.8)	7 [4-8]	148	110 (74.3)	6 [3-8]	102	-0.75 (2.00)*
Males	131	100 (76.3)	6 [3-7]	135	108 (80.0)	6 [3-7]	95	-0.44 (1.96)*


IQR: interquartile range, SD: standard deviation, ADL: activities of daily living, IADL: instrumental activities of daily living. *Statistically significant.

Table 3 shows the cross-sectional analyses of the association of HGS and muscle mass with dependency in ADL and IADL at admission. Lower HGS was significantly associated with ADL dependency in both females and males. Lower SMM, SMM% and SMI were significantly associated with ADL dependency in males, but not in females. Lower HGS was significantly associated with IADL dependency in males, but not in females. SMM, SMM% and SMI were not associated with IADL.

**Table 3:** Association of muscle measures with dependency in Activities of Daily Living and Instrumental Activities of Daily Living at admission, stratified for sex

		**ADL dependency**			**IADL dependency**		
		**N**	**OR (95% C.I.)**	**P-value**	**N**	**OR (95% C.I.)**	**P-value**
*Females*							
HGS, kg	Model 1	157	0.92 (0.86 - 0.98)	0.015	111	0.95 (0.87 - 1.04)	0.290
	Model 2		0.90 (0.83 - 0.96)	0.003		0.93 (0.84 - 1.02)	0.126
SMM, kg	Model 1	157	1.05 (0.96 - 1.15)	0.284	111	1.02 (0.91 - 1.14)	0.725
	Model 2		1.01 (0.92 - 1.11)	0.833		0.97 (0.86 - 1.10)	0.644
SMM, %	Model 1	157	0.95 (0.90 - 1.01)	0.096	111	0.96 (0.90 - 1.03)	0.288
	Model 2		1.00 (0.92 - 1.08)	0.915		0.98 (0.89 - 1.08)	0.710
SMI, kg/m^2^	Model 1	157	1.17 (0.90 - 1.53)	0.246	111	1.06 (0.77 - 1.45)	0.723
	Model 2		1.01 (0.75 - 1.37)	0.925		0.89 (0.62 - 1.28)	0.529
*Males*							
HGS, kg	Model 1	158	0.93 (0.89 - 0.97)	0.001	112	0.94 (0.89 - 0.99)	0.023
	Model 2		0.94 (0.90 - 0.98)	0.007		0.94 (0.89 - 1.00)	0.057
SMM, kg	Model 1	158	0.86 (0.79 - 0.93)	<0.001	112	0.95 (0.88 - 1.03)	0.193
	Model 2		0.86 (0.80 - 0.94)	<0.001		0.96 (0.88 - 1.04)	0.303
SMM, %	Model 1	158	0.93 (0.87 - 1.00)	0.040	112	0.98 (0.90 - 1.08)	0.706
	Model 2		0.87 (0.80 - 0.95)	0.002		0.96 (0.87 - 1.07)	0.492
SMI, kg/m^2^	Model 1	158	0.60 (0.46 - 0.79)	<0.001	112	0.82 (0.60 - 1.11)	0.196
	Model 2		0.61 (0.46 - 0.80)	<0.001		0.85 (0.61 - 1.18)	0.338


Logistic regression analyses. ADL: activities of daily living, IADL: instrumental activities of daily living, OR: odds ratio, CI: confidence interval, HGS: handgrip strength, SMM: skeletal muscle mass, SMI: skeletal muscle index. Model 1: adjustment for age. Model 2: model 1 and multimorbidity and, weight and height (for HGS), or fat mass (for SMM and SMI), or weight (for SMM%). Bold values are statistically significant.

Table 4 shows the association of HGS and muscle mass at admission with change in ADL and IADL scores between admission and three months after discharge. Lower HGS and SMM% at admission were significantly associated with a decrease in ADL score indicating a relative increase of dependency in males, not in females. SMM and SMI were not associated with change in ADL score. Lower SMM and SMI showed a trend with a decrease in ADL scores in males. No association was found with change in IADL score.

**Table 4:** Association of muscle measures at admission with change in activities of daily living and instrumental activities of daily living scores, stratified by sex

		**Change in ADL score**			**Change in IADL score**		
		**N**	**ß (95% C.I.)**	**P-value**	**N**	**ß (95% C.I.)**	**P-value**
*Females*							
HGS, kg	Model 1	125	0.03 (-0.03 - 0.08)	0.320	83	0.00 (-0.09 - 0.10)	0.952
	Model 2		0.04 (-0.01 - 0.09)	0.144		-0.01 (-0.12 - 0.09)	0.832
SMM, kg	Model 1	125	-0.01 (-0.07 - 0.05)	0.739	83	0.06 (-0.06 - 0.17)	0.335
	Model 2		0.00 (-0.07 - 0.07)	0.933		0.07 (-0.05 - 0.20)	0.235
SMM, %	Model 1	125	0.01 (-0.04 - 0.05)	0.821	83	0.05 (-0.03 - 0.13)	0.191
	Model 2		-0.01 (-0.07 - 0.04)	0.673		0.07 (-0.03 - 0.17)	0.161
SMI, kg/m^2^	Model 1	125	0.00 (-0.19 - 0.20)	0.980	83	0.14 (-0.21 - 0.49)	0.437
	Model 2		0.05 (-0.17 - 0.27)	0.669		0.22 (-0.17 - 0.61)	0.266
*Males*							
HGS, kg	Model 1	116	0.03 (0.00 - 0.06)	0.027	79	0.01 (-0.04 - 0.06)	0.675
	Model 2		0.03 (0.00 - 0.06)	0.021		0.01 (-0.05 - 0.06)	0.851
SMM, kg	Model 1	116	0.04 (-0.01 - 0.08)	0.093	79	0.05 (-0.03 - 0.12)	0.210
	Model 2		0.04 (-0.01 - 0.08)	0.087		0.05 (-0.03 - 0.12)	0.228
SMM, %	Model 1	116	0.05 (0.00 - 0.09)	0.030	79	0.01 (-0.07 - 0.08)	0.894
	Model 2		0.06 (0.01 - 0.11)	0.020		0.03 (-0.06 - 0.11)	0.513
SMI, kg/m^2^	Model 1	116	0.16 (-0.02 - 0.33)	0.078	79	0.22 (-0.09 - 0.53)	0.169
	Model 2		0.17 (0.00 - 0.35)	0.053		0.21 (-0.11 - 0.52)	0.192


Linear regression analyses. ADL: activities of daily living, IADL: instrumental activities of daily living, OR: odds ratio, CI: confidence interval, HGS: handgrip strength, SMM: skeletal muscle mass, SMI: skeletal muscle index. Positive changes ADL and IADL scores indicate an increase in independency and negative changes in ADL or IADL scores indicate a decrease in independency. Model 1: adjustment for age and admission ADL/IADL score. Model 2: model 1 and multimorbidity and weight and height (for HGS), or fat mass (for SMM and SMI), or weight (for SMM%). Bold values are statistically significant.

## Discussion

In an observational prospective inception cohort study of hospitalized patients aged 70 years and older lower hand grip strength (HGS) was associated with ADL dependency at admission. Muscle mass, i.e. relative and absolute skeletal muscle mass and skeletal muscle mass index were associated with ADL at admission, but in males only. Both lower HGS and relative skeletal mass were associated with an increase of ADL dependency at three months after discharge, but only in males. Only HGS at admission was associated with IADL at admission and only in males.

HGS was consistently associated with ADL dependency. HGS values in our population were similar to sarcopenic community dwelling subjects and acutely hospitalized subjects (28, 29) and lower compared to geriatric outpatients (27). HGS in female patients was comparable to much older individuals of the general population (aged 89 years and older) (30) and chronically ill patients (31). Values of muscle mass measures similar to community dwelling older subjects (32) and geriatric outpatients (33).

Associations of muscle measures with ADL dependency are in line with previous findings in community dwelling older adults (26, 34, 35). Other studies have found muscle strength to be associated with both ADL and IADL (36). Our study showed only a trend for handgrip strength and IADL in males. Stronger associations of muscle measures with ADL might be explained by the fact that Katz ADL and Lawton and Brody IADL score cover different domains of ADL (37): the Katz ADL involves basic ADL functions and IADL encompasses more complex tasks in which cognition plays a more dominant role (38, 39, 40). Muscle mass, as an obvious contributing factor to muscle strength (41) was found to be relatively preserved in the present cohort and associated with ADL in males only. Results are in line with the notion that muscle strength may be influenced by other factors than muscle quantity alone (42) and that muscle mass and muscle strength are different constructs (43), which relate to clinical outcomes in different ways (44, 45). An apparent sex- specificity in this respect requires further attention.

In the present study, a significant decline three months after admission was found for the IADL scores, implying that IADL is more sensitive to change than basic ADL activities after hospital discharge in older patients (37).

In a previous cohort study of 492 older hospitalized subjects, 54% was found to be independent in ADL before admission and 44% 3 months after admission (46). One third of the patients suffered a functional decline defined of at least one point loss on the Katz score three months after discharge. A decline in independency after hospitalization is further substantiated by a previous study in which most patients became more dependent after hospital admission and some did not return to their pre-hospitalization dependency (46). The question whether hospitalization by itself is a risk factor for ALD decline is difficult to answer without comparison to trajectories of ADL decline in similar but not hospitalized subjects. In a previous study, muscle measures were not found to change during hospitalization (47) although immobilization during hospital stay was found to have acute negative impact on muscle mass and strength (48), which might be further substantiated after discharge.

### Strength and limitations

EMPOWER is a large prospective inception cohort study of older adults admitted to various specialisms minimizing selection bias and ensuring diversity, representative for an older hospital population. Muscle mass parameters were measured by use of portable BIA devices, which is susceptible to hydration state which was as much as possible controlled for. Patients who were expected to be discharged within 24 hours of admission were not included due to the short stay, which could have caused selection bias excluding the healthier patients. The used ADL and IADL scales have ceiling effects reducing sensitivity for changes and potentially interfering with addressed associations.

## Conclusion

Muscle measures at hospital admission in older patients are related to ADL dependency at admission and change in ADL dependency three months after discharge, however, these associations are sex specific. Taken aforementioned findings into account, HGS may be useful to predict outcome after hospital admission. Further research into trajectories of ADL dependency, before, during and after hospitalization are required to further pinpoint risk factors, patients at risk and identification and allocation of preventive measures.
